# Supplementary material for: Nucleoside analogue activators of cyclic AMP-independent protein kinase A of Trypanosoma
Source: Nat Commun. 2019 Mar 29;10:1421. doi: 10.1038/s41467-019-09338-z (PMC6440977; doi:10.1038/s41467-019-09338-z)
Supplement: Supplementary file 1 — Supplementary Information [file 41467_2019_9338_MOESM1_ESM.pdf]

Supplementary Information

Bachmaier et al., Nucleoside analog activators of cyclic AMP-independent protein kinase A of *Trypanosoma*

Supplementary Figure 1

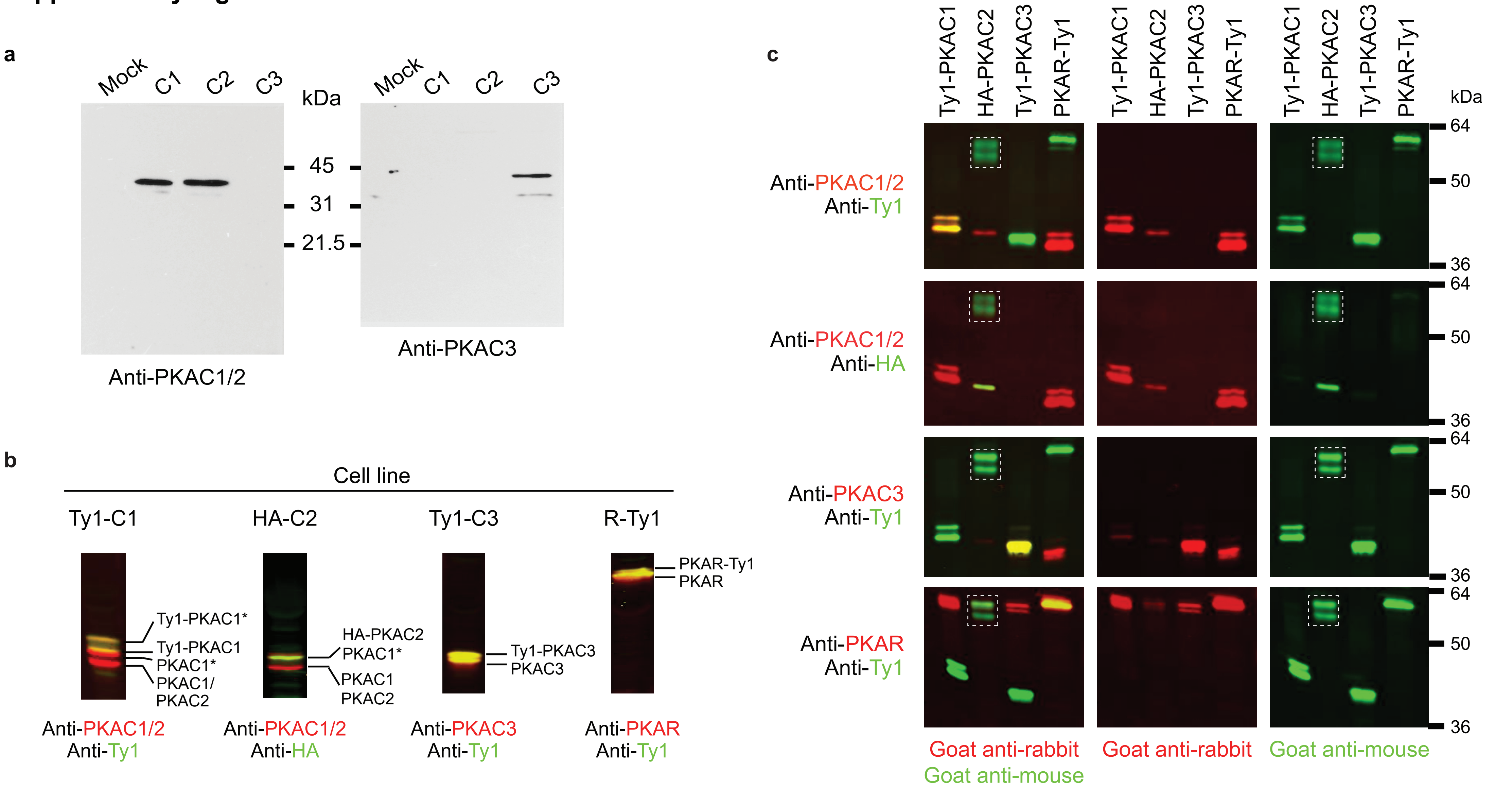

Characterization of PKA holoenzyme complexes

(a) Specificity of PKAC antibodies: *T. brucei* PKAC1, PKAC2 and PKAC3 were transiently expressed in human 293 cells as GST fusion proteins and probed on Western blots with anti-PKAC1/2 (left) or anti-PKAC3 (right) affinity purified rabbit sera. Cells transfected with the empty vector were included as control (Mock).

(b) Analysis of *T. brucei* cell lines expressing epitope-tagged PKA subunits. PKAC1 and PKAC2 were expressed as Ty1- or HA-epitope tag fusion, respectively, from their endogenous loci, whereas PKAC3 and PKAR were ectopically overexpressed as Ty1-tag fusions. The Western blots were probed with the respective PKA subunit and epitope tag antibodies. PKAC1* and Ty1-PKAC1* indicate a modified form of PKAC1 with higher apparent molecular mass. Source data are provided as a Source Data file.

(c) PKA holoenzyme complexes: Ty1-PKAC1, HA-PKAC2, Ty1-PKAC3 and PKAR-Ty1 were pulled down by epitope tag antibodies anti-Ty1 or anti-HA. The beads were analyzed on four parallel blots and each blot was probed with a different PKA antibody together with an epitope tag antibody, as indicated. Secondary antibodies were Alexa Fluor® 680 goat anti-rabbit (red, center panels), or IRDye 800CW goat anti-mouse (green, right panels) or both secondary antibodies (left panels). Note that the goat anti-mouse also recognizes the heavy chain of anti-HA IgG (white dashed square), since anti-HA was not cross-linked to the sepharose beads.

Supplementary Figure 2

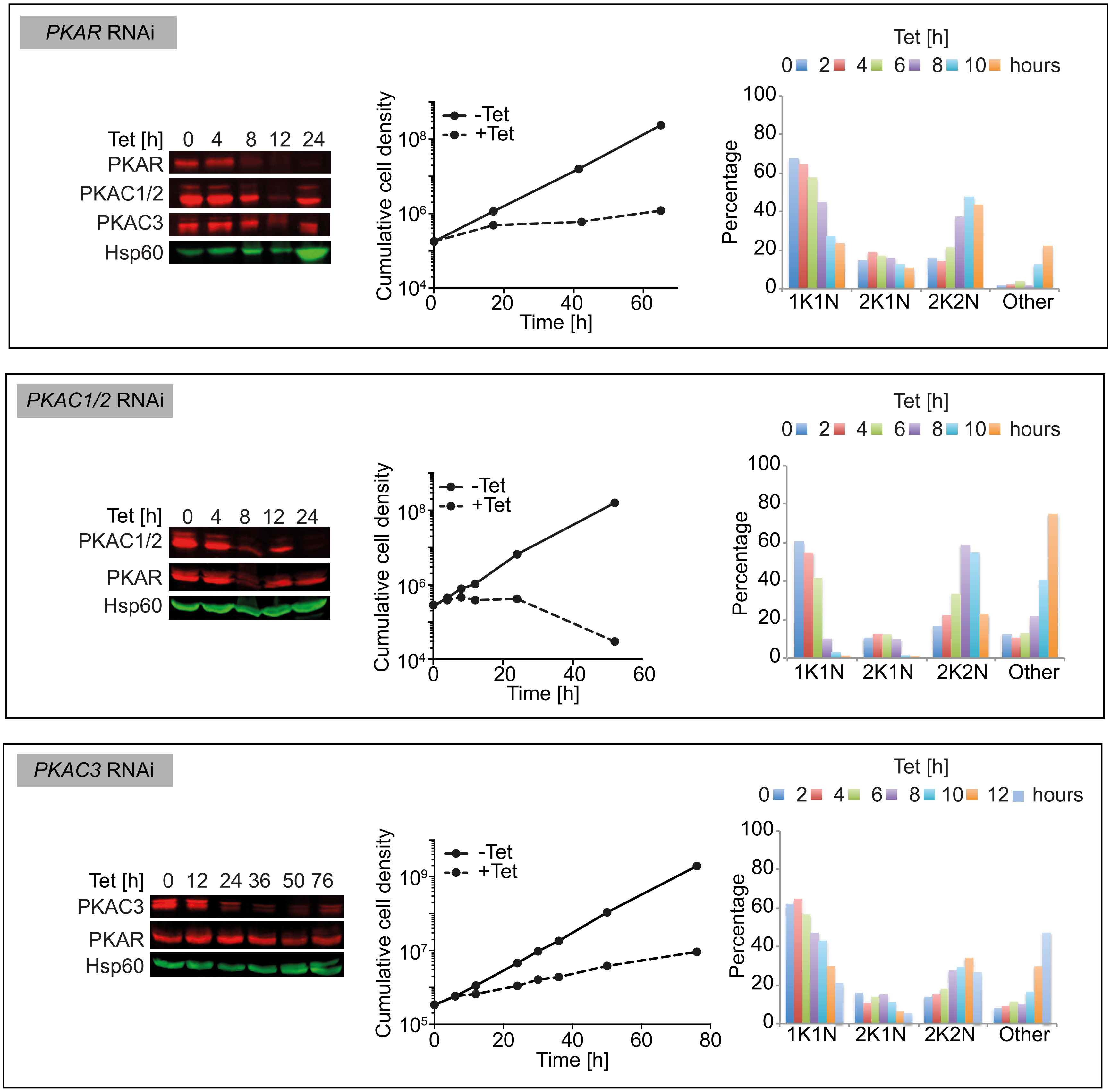

Growth and cell division phenotypes upon inducible RNAi depletion of PKA subunits in *T. brucei*
RNAi efficiency for PKAR (top), PKAC1/2 (middle) or PKAC3 (bottom) was determined by Western blot analysis at the indicated time points after induction of RNAi with 1 µg ml^{-1} tetracycline (Tet). Loading control is Hsp60. The cumulative cell growth was monitored and cell cycle stages were determined by microscopic counting ((>100 cells per time point) kinetoplast (K) / nucleus (N) configurations). For analysis of K/N configurations, nuclear DNA and the DNA of the single mitochondrion (kinetoplast) are both stained with DAPI. Since the kinetoplast divides prior to the nucleus, three cell cycle phases can be distinguished: cells with one kinetoplast (K) and one nucleus (N) (1K1N; G1 phase of cell cycle), cells with two kinetoplasts and one nucleus (2K1N; S to early M phase of cell cycle) and cells with two kinetoplasts and two nuclei (2K2N; between mitosis and cytokinesis). A logarithmically growing trypanosome population contains around 70% 1K1N cells and around 15% of each 2K1N and 2K2N cells. Aberrant configurations like multinucleated (>2N) cells are scored as “other”. Source data are provided as a Source Data file.

Supplementary Figure 3

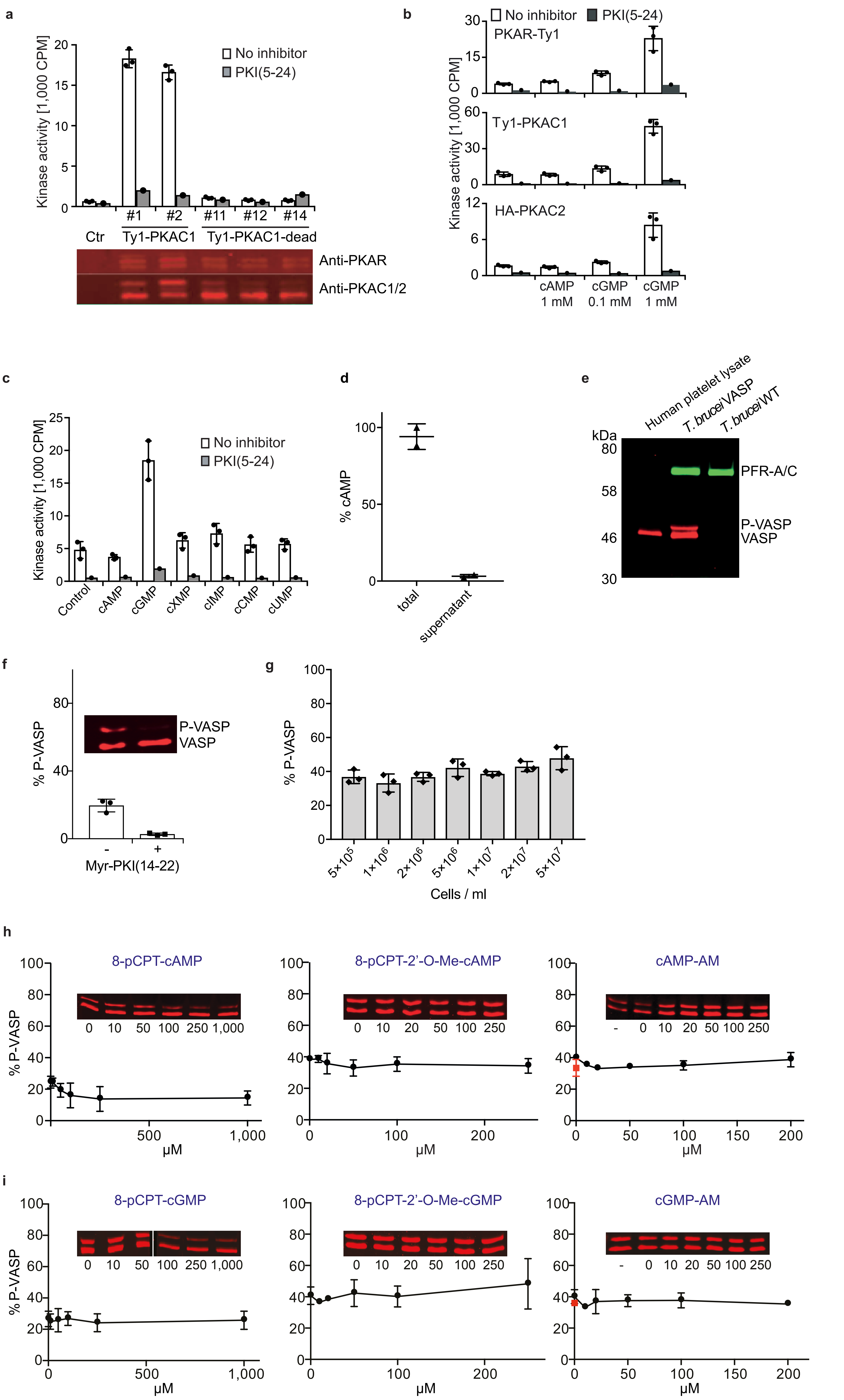

Kinase activity in response to cyclic nucleotides and analogs

(a) In vitro kinase assays of immuno-purified *T. brucei* PKA holoenzymes. PKA holoenzyme complexes were pulled down from trypanosome cell lines expressing Ty1-PKAC1 (independent clones #1, #2) or the N165->A kinase dead mutant (Ty1-PKAC1-dead, independent clones #11, #12, #14) for assay of kemptide phosphorylation in the presence or absence of 5 µM PKI(5-24). Wild type cells were included for the negative control pull down (Ctr). Equivalent amounts of kinase in all samples were verified by Western blots using the indicated antisera.

(b) In vitro kinase assays as in (a) in the presence or absence of cAMP or cGMP as indicated. PKA holoenzyme was immunopurified via PKAR-Ty1 (upper panel) or Ty1-PKAC1 (middle panel) or HA-PKAC2 (lower panel).

(c) In vitro kinase assays as in (a) in the presence or absence of 1 mM of the indicated cyclic nucleotides (cXMP: xanthosine-3',5'-cyclic monophosphate; cIMP: inosine-3',5'-cyclic monophosphate; cCMP: cytidine-3',5'-cyclic monophosphate; cUMP: uridine -3',5'-cyclic monophosphate). PKA holoenzyme was pulled down via Ty1-PKAC1. Columns in (a)-(c) represent the mean ± SD of three independent replicates. For each condition, a single control with 5 µM of PKA-specific peptide inhibitor PKI(5-24) was included (grey columns).

(d) Fraction of cAMP detected in cell supernatant versus total cell lysate upon 15 min treatment with 10 µM CpdA (mean ± range of n = two technical replicates).

(e) Transgenic expression of the human PKA substrate VASP in *T. brucei* (cell line *T. brucei* VASP). The Western blot loaded with whole cell lysates shows both the phosphorylated (P-VASP) and unphosphorylated (VASP) forms of VASP distinguishable by an electrophoretic mobility shift. Human platelet lysate and whole cell lysate of parental *T. brucei* MiTat 1.2 cells (*T. brucei* WT) serve as controls. Loading control for the *T. brucei* cell lysates is PFR-A/C.

(f-i) In vivo PKA reporter assay using cell line *T. brucei* VASP **(f)** in the presence or absence of 200 µM membrane-permeable myr-PKI(14-22) for 15 min prior to lysis or **(g)** at increasing cell densities as indicated (30 min incubation) or **(h)** upon treatment with membrane-permeable cAMP analogues or **(i)** membrane-permeable cGMP analogues at the indicated concentrations and treatment for 15 min prior to lysis. For compounds dissolved in DMSO, the solvent control corresponds to the '0 µM' sample. The untreated controls are indicated (-, ■) where applicable. The fraction of phosphorylated VASP ((P-VASP/(P-VASP+VASP)) ×100) that is a proxy of kinase activity was quantified from Western blots (one representative shown as inset). The plotted values represent mean ± SD of three independent replicates.The Source data to (a-d) and (f-i) are provided as a Source Data file.

Supplementary Figure 4

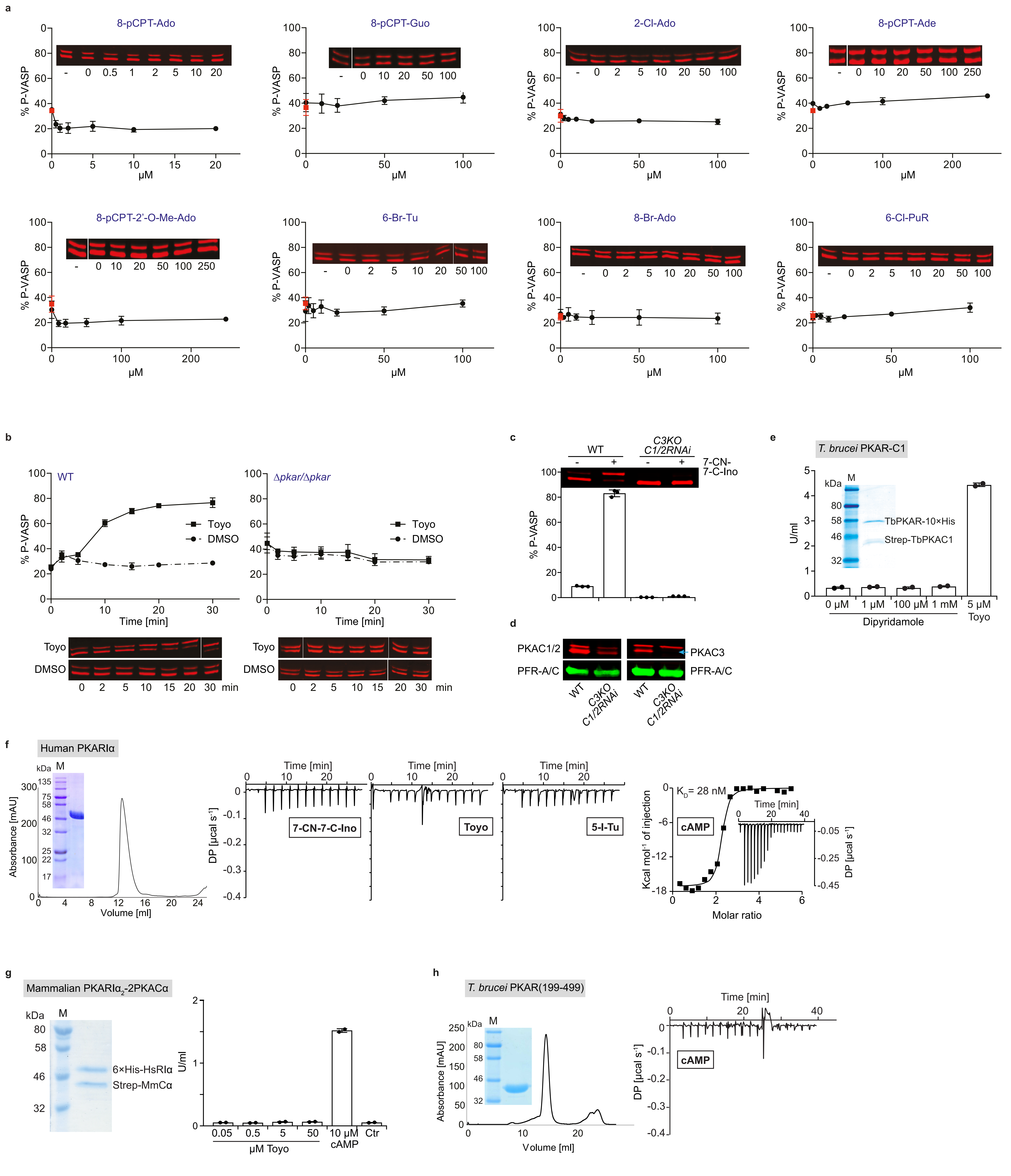

Kinase activation and binding of selected nucleoside analogs

(a) Dose response for in vivo kinase assay: VASP phosphorylation was quantified from Western blots after incubation of trypanosomes with the indicated compounds for 15 min. The solvent control (DMSO) corresponds to the '0 µM' sample; untreated cells serve as negative control (-,■).

(b) Time course for in vivo kinase assay: VASP phosphorylation was quantified in VASP expressing trypanososomes with wild type (WT) or mutant background with homozygous deletion of *PKAR* (*Δpkar/Δpkar*) in the presence of 250 nM toyocamycin (Toyo, ■) or 1% DMSO (●) over 30 min.

(c) In vivo PKA reporter assay in VASP expressing trypanosomes with wild type (WT) or mutant background with homozygous deletion of *PKAC3* and inducible RNAi repression of *PKAC1/2* (*C3KO C1/2 RNAi*). After 18 h of tetracycline repression of *PKAC1/2*, the inducer 7-CN-7-C-ino (4 µM) or 1% DMSO were added for 15 min.

(d) Western blot control of PKAC expression (anti-PKAC1/2 left; anti-PKAC3 right) in the cell lines from (c) with PFR-A/C as loading control.

Error bars in (a-c) represent SD of three independent replicates and one representative Western blot is shown for each experiment.

(e) Tandem affinity purification of TbPKA holoenzyme (Coomassie-stained protein gel). PKAR-10×His and Strep-PKAC1 were co-expressed in *L. tarentolae* (LEXSY system). Dipyridamole was tested in kinase assays with purified holoenzyme with Toyo as positive control. Error bars give the range of duplicate assays from a representative experiment (two biological replicates).

(f) Purification of 50 kDa human PKARIα monomer recombinantly expressed in *E. coli*. The size-exclusion chromatogram and the Coomassie-stained protein from the peak fraction (inset) are shown. Binding of 7-CN-7-C-Ino, Toyo, 5-I-Tu and cAMP (positive control) to this protein was assayed by ITC . The power differential (DP) traces are shown for all compounds (representative of 2 independent replicates), a binding curve can only be calculated for cAMP.

(g) Tandem affinity purification of mammalian PKARIα_2-2PKACα holoenzyme (Coomassie-stained protein gel). 6×His-HsPKARα and Strep-MmPKACα were co-expressed in *E. coli*. Toyo was tested in kinase assays with purified holoenzyme with cAMP as positive control and DMSO as solvent control (ctr). Error bars give the range of duplicate assays from a representative experiment (two biological replicates).

(h) Purification of 35 kDa monomer of TbPKAR(199-499) recombinantly expressed in *E. coli*. The size-exclusion chromatogram and the Coomassie-stained protein from the peak fraction (inset) are shown. Binding of cAMP to this protein was assayed by ITC. The power differential (DP) trace is shown (representative of 2 independent replicates).

Source data are provided as a Source Data file.

Supplementary Figure 5

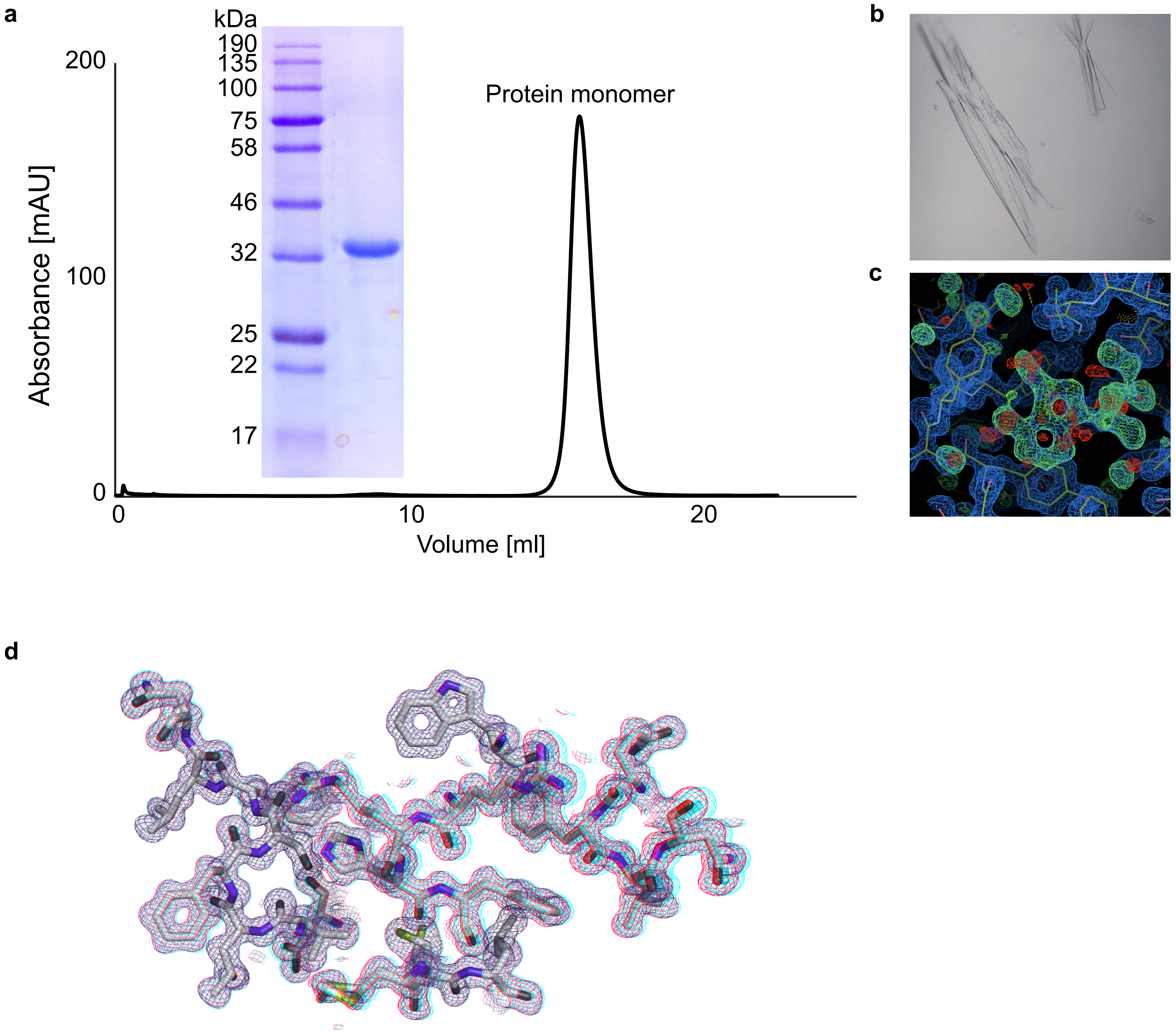

Co-crystallization of *T. cruzi* PKAR(200-503) with 7-CN-7-C-Ino
(a) *T. cruzi* PKAR(200-503) purified from *E. coli* was re-folded in the presence of 1 mM 7-CN-7-C-Ino followed by size-exclusion chromatography. The protein eluted as monomer as judged from the elution volume from a calibrated column. The inset shows a Coomassie-stained SDS-PAGE of the purified protein.
(b) Protein crystals were observed after one week at 4°C with a reservoir solution made of 17% PEG 6,000 and 0.2 M calcium acetate.
(c) Fo-Fc electron density map (CNB-B pocket) calculated after structure solution but prior to fitting of the ligand inside the map.
(d) Portion of the polypeptide chain showing a 3D anaglyph stereo image of the 2Fo-Fc electron density map at 1σ cutoff.

Supplementary Figure 6

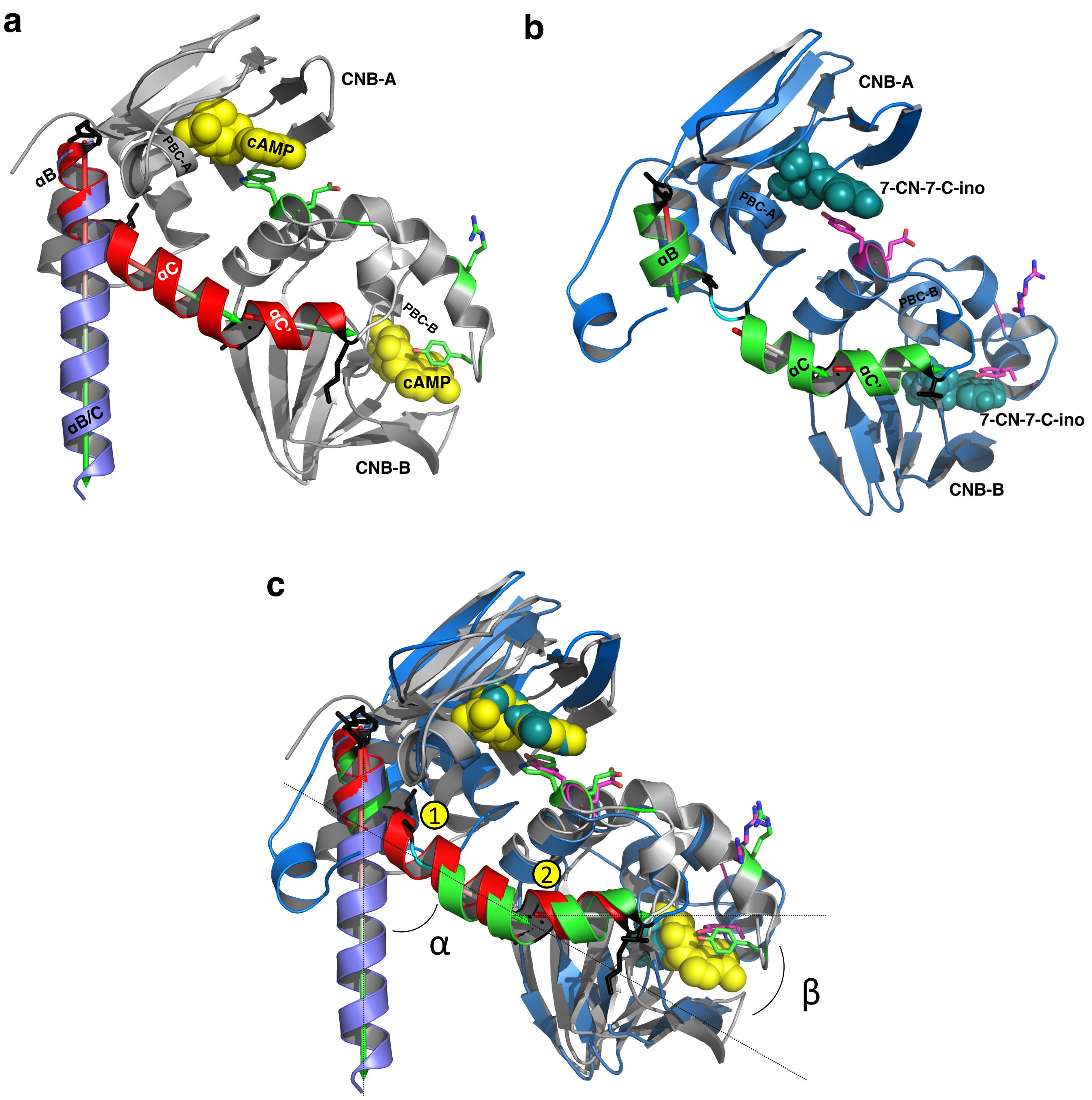

Structural comparison of the αB/C helix element

(a) Helices αB, αC and αC' (red) of bovine PKARIα (PBD 1RGS, grey) bound to cAMP (yellow). The αB/C helix of mammalian PKAR in the holoenzyme conformation (PDB 2QCS) is overlayed for illustration (purple). Amino acids important for the activation mechanism are highlighted in green. The orientation vectors inside the helices are color coded red to green to indicate N to C terminal orientation.

(b) Helices αB, αC and αC' (green) of *T. cruzi* PKAR (blue) bound to 7-CN-7-C-Ino (teal).

(c) Overlay of (a) and (b). The angles α and β produced by the 3 sections of the αB/C helix have previously been calculated for PKARIα (55º and 30º) and are perfectly maintained in the TcPKAR structure with 55º and 28º, respectively. Inflection points are indicated (positions 1 and 2) corresponding to L233 and Y244 of bovine PKARIα. In TcPKAR position 1 is a small loop region formed by the sequence MGTA while position 2 corresponds to Y355.

Supplementary Figure 7

a

TcPKAR CNB-like A

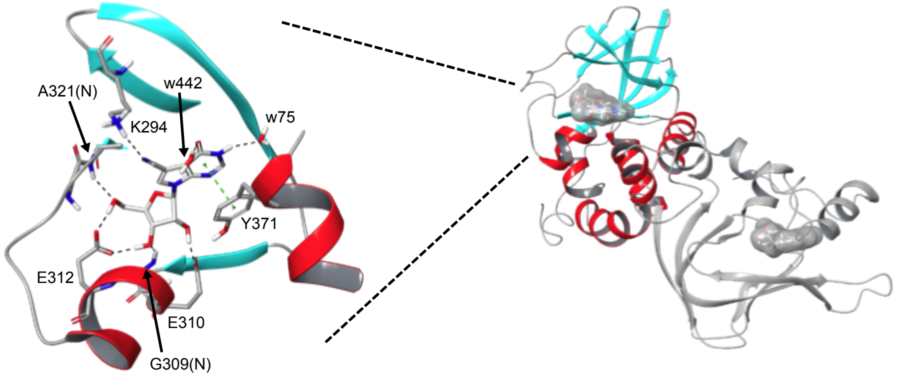

7-CN-7-C-Ino
GE = -99.090

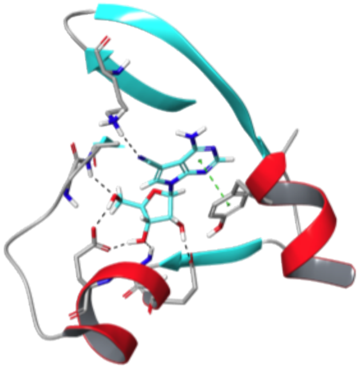

Toyo
GE = -84.974

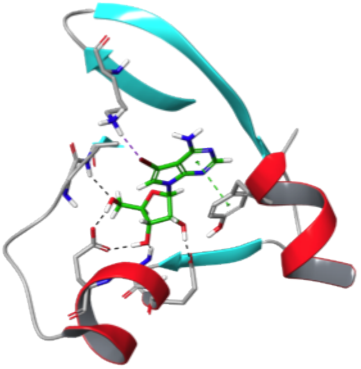

5-Br-Tu
GE = -79.983

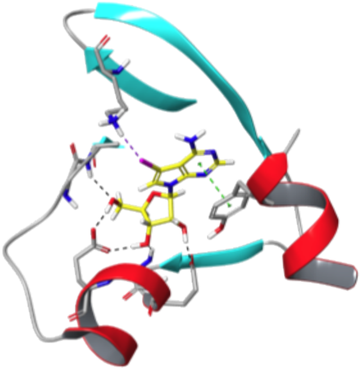

5-I-Tu
GE = -78.521

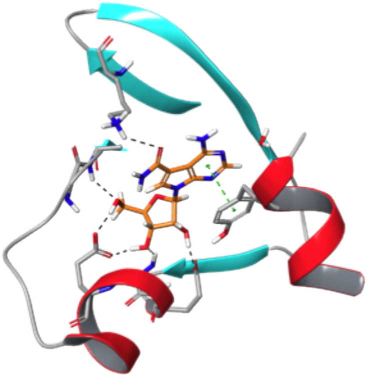

Sangivamycin
GE = -70.449

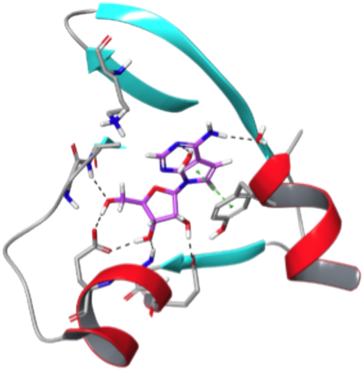

Tu
GE = -69.909

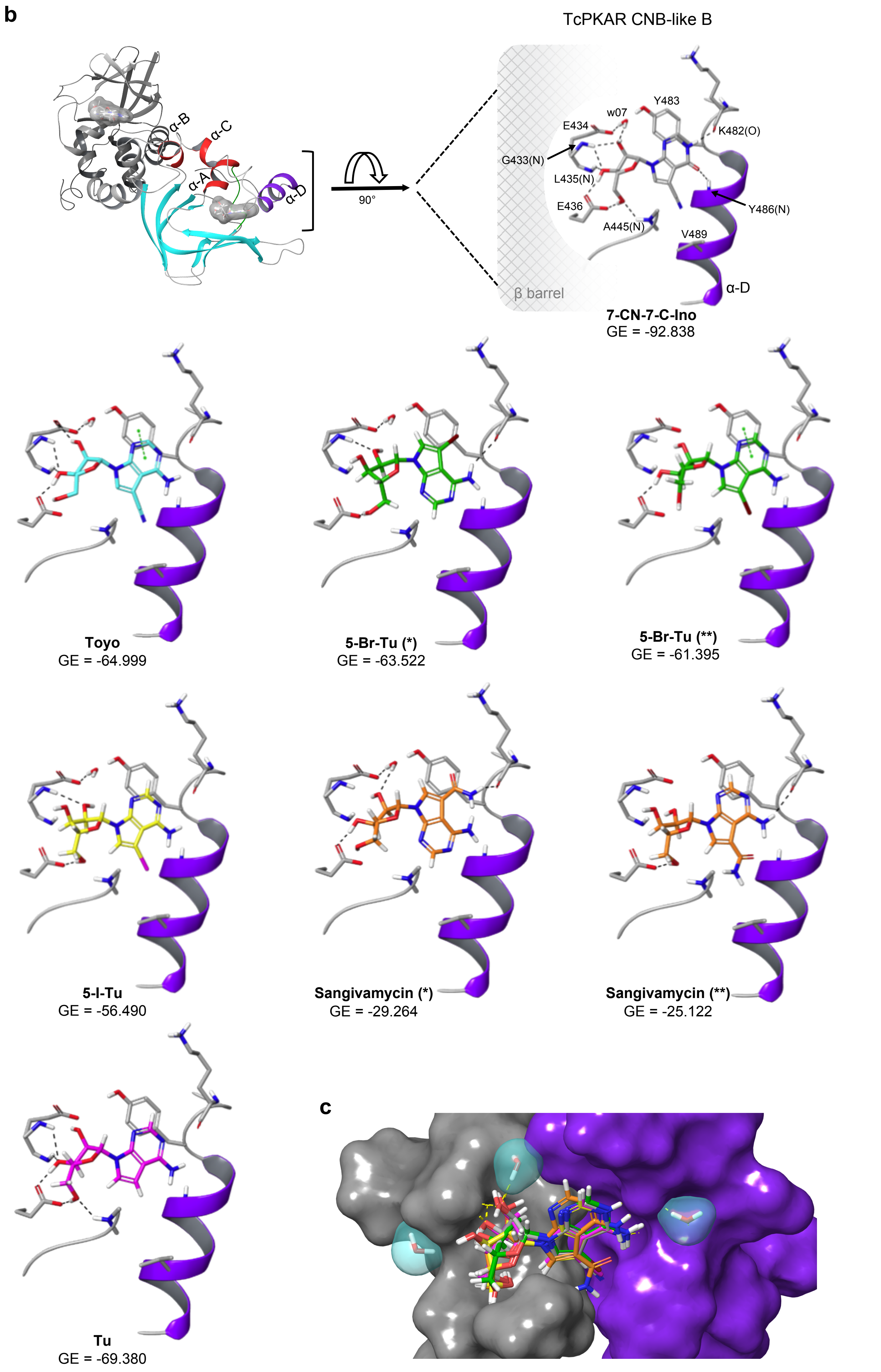

Computational docking of 7-deaza nucleoside activators

The activators listed in Table 1 were docked to **(a)** the CNB-like A and **(b)** CNB-like B site, respectively, of the *T. cruzi* PKAR crystal structure using Glide. For each compound the best pose ranked by Glide Emodel is shown. In the CNB-like B site, the best scoring poses for 5-Br-Tu and Sangivamycin have their 6-membered ring flipped downwards and are marked with a (*). The next best pose displaying this ring flipped up, and therefore in the same orientation as 7-CN-7-C-Ino, is marked with (**). The α-D helix at the C-terminus is displayed in purple.

(c) Surface representation of the helix α-D (purple) and the beta barrel (grey) of the CNB-like B site. A hydrophobic pocket is formed at the interface of those two subdomains (side chains of V444, V489, Y485, Y486). Five of the six docked analogs are displayed for comparison of their side groups in the hydrophobic cavity. Carbons of 7-CN-7-C-Ino, 5-Br-Tu(**), 5-I-Tu, Tu and Sangi are coloured in grey, green, yellow, magenta and orange, respectively.

Supplementary Figure 8

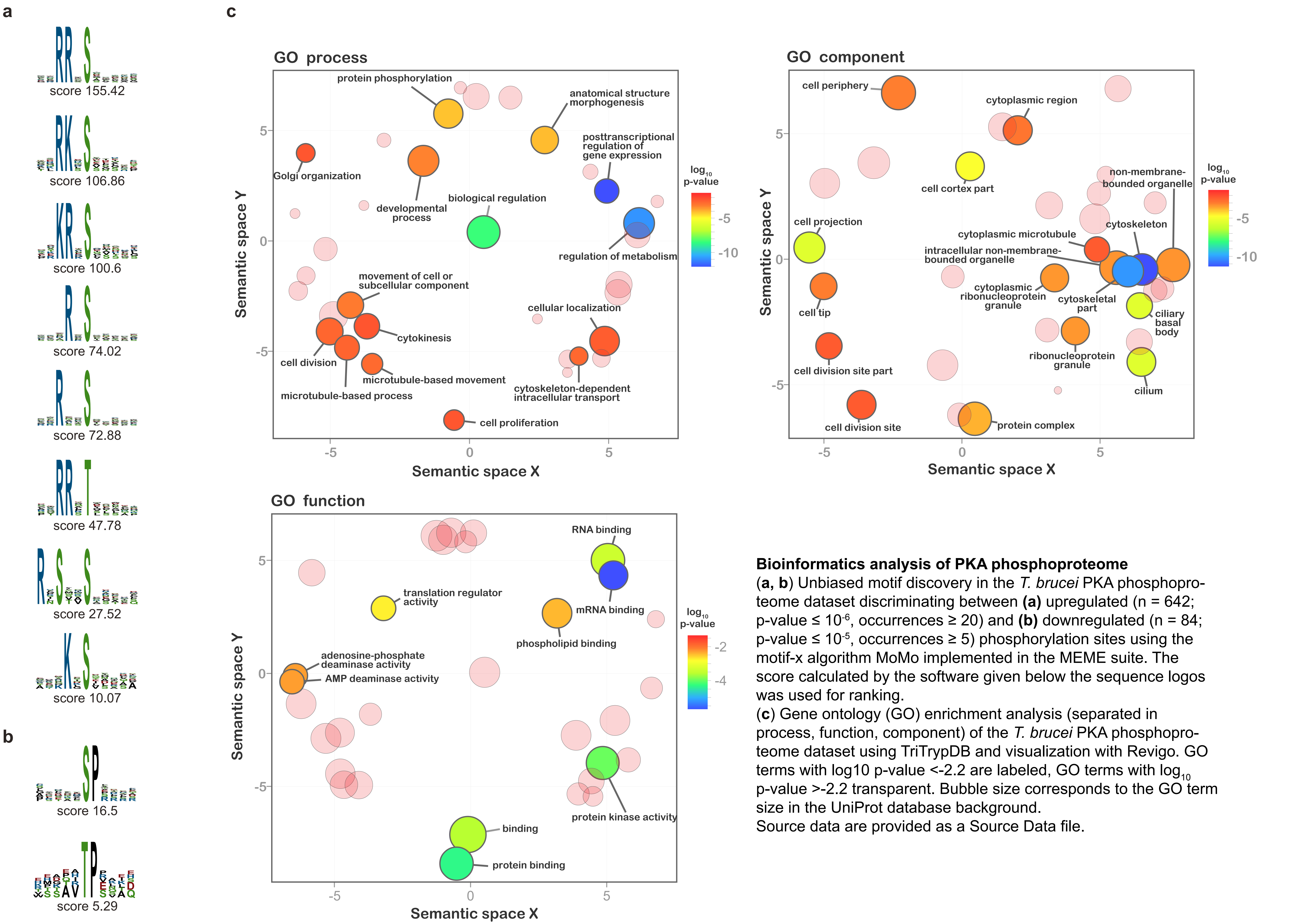

Bioinformatics analysis of PKA phosphoproteome
(**a, b**) Unbiased motif discovery in the *T. brucei* PKA phosphoproteome dataset discriminating between **(a)** upregulated (n = 642; p-value ≤ 10^{-6}, occurrences ≥ 20) and **(b)** downregulated (n = 84; p-value ≤ 10^{-5}, occurrences ≥ 5) phosphorylation sites using the motif-x algorithm MoMo implemented in the MEME suite. The score calculated by the software given below the sequence logos was used for ranking.
(**c**) Gene ontology (GO) enrichment analysis (separated in process, function, component) of the *T. brucei* PKA phosphoproteome dataset using TriTrypDB and visualization with Revigo. GO terms with log10 p-value <-2.2 are labeled, GO terms with $\log_{10}$ p-value >-2.2 transparent. Bubble size corresponds to the GO term size in the UniProt database background.
Source data are provided as a Source Data file.

Supplementary Figure 9

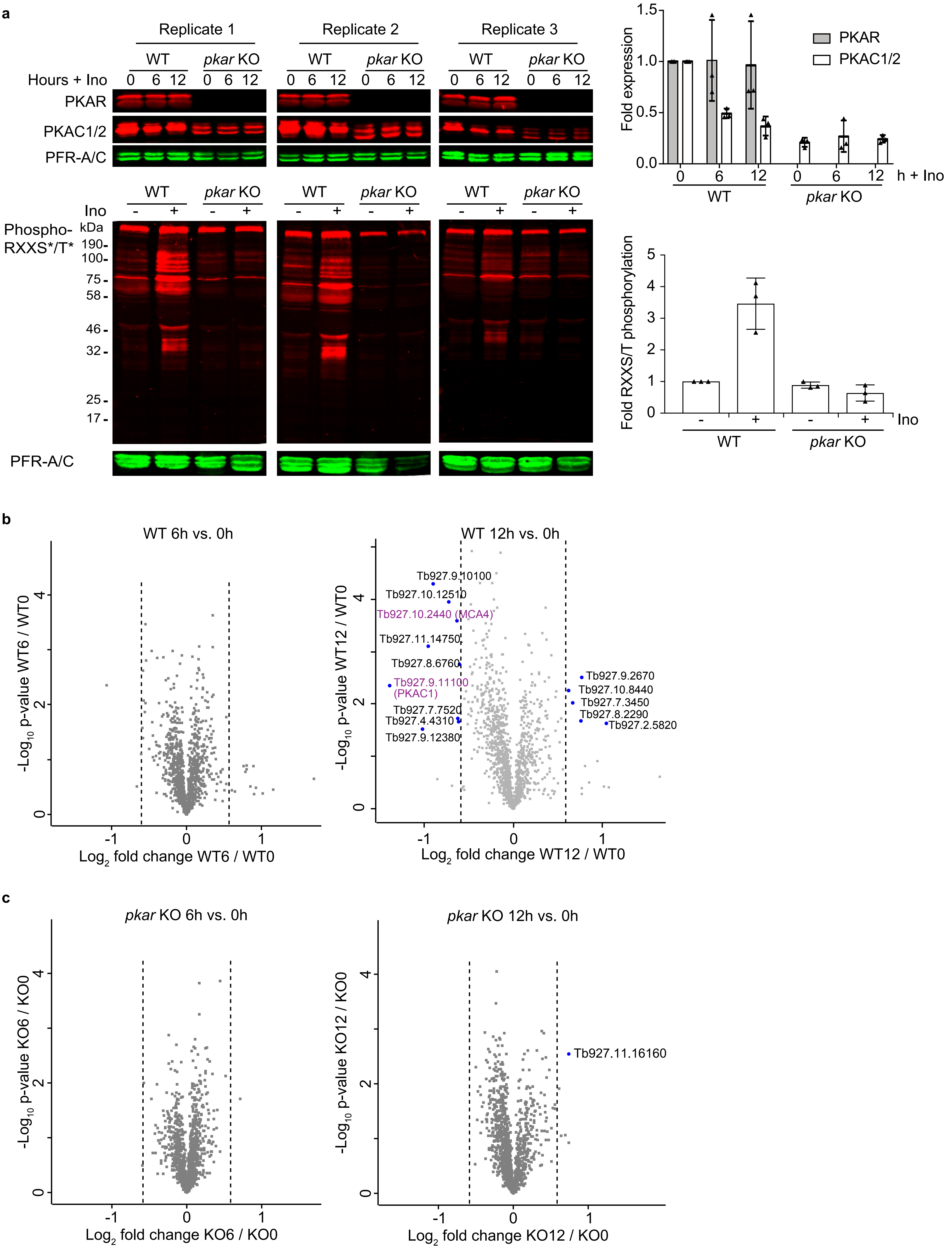

PKA substrates and target proteins

(a) Samples prepared for quantitative proteome analysis: PKAR and PKAC1/2 protein levels (top Western panels, red) and RXXS*/T* phosphorylation (bottom Western panels, phospho-specific anti-RXXS*/T*, red) are quantified as summarized in the column charts next to the Western blots. The scanned values were normalized to the loading control (PFR-A/C, green) and the untreated wild type sample was set to 1. Wild type (WT) and Δ*pkar*/Δ*pkar* (pkar KO) cells were treated with 7-CN-7-C-Ino (Ino) for the indicated time (PKAR/PKAC1 expression) or for 10 min (RXXS*/T* phosphorylation). Error bars represent SD of three independent experiments.

(b, c) Volcano plot representations of proteins quantified by label-free proteomics in WT (**b**) or Δ*pkar*/Δ*pkar* (*pkar* KO) cells (**c**). Proteins are plotted according to p-value and fold change caused by a 6-hour or 12-hour treatment with 7-CN-7-C-Ino as indicated. Proteins changing >1.5 fold (vertical dashed lines) with a p-value of <0.05 (Welch's t-test, two-sided) are labeled as blue dots with their TriTrypDB IDs. Source data are provided as a Source Data file.

Supplementary Table 1. Compounds used in this study

Compound	Abbreviation
8-(4-Chlorophenylthio)adenosine-3',5'-cyclic monophosphate	8-pCPT-cAMP
8-(4-Chlorophenylthio)-2'-O-methyladenosine-3',5'-cyclic monophosphate	8-pCPT-2'-O-Me-cAMP
8-(4-Chlorophenylthio)guanosine-3',5'-cyclic monophosphate	8-pCPT-cGMP
8-(4-Chlorophenylthio)-2'-O-methylguanosine-3',5'-cyclic monophosphate	8-pCPT-2'-O-Me-cGMP
Adenosine-3',5'-monophosphate, acetoxymethyl ester	cAMP-AM
Guanosine-3',5'-cyclic monophosphate, acetoxymethyl ester	cGMP-AM
Tubercidin,<br>7-Deazaadenosine	Tu
5-Bromotubercidin,<br>7-Bromo-7-deazaadenosine	5-Br-Tu
5-Iodotubercidin,<br>7-Iodo-7-deazaadenosine	5-I-Tu
Toyocamycin,<br>7-Cyano-7-deazaadenosine	Toyo
Sangivamycin,<br>7-Carboxamido-7-deazaadenosine	
6-Bromotubercidin,<br>8-Bromo-7-deazaadenosine	6-Br-Tu
7-Cyano-7-deazainosine	7-CN-7-C-Ino
8-(4-Chlorophenylthio)adenosine	8-pCPT-Ado
8-(4-Chlorophenylthio)-2'-O-methyladenosine	8-pCPT-2'-O-Me-Ado
8-(4-Chlorophenylthio)adenine	8-pCPT-Ade
8-(4-Chlorophenylthio)guanosine	8-pCPT-Guo
8-Bromoadenosine	8-Br-Ado
2-Chloroadenosine	2-Cl-Ado
6-Chloropurine riboside	6-Cl-PuR
Dipyridamole	Dip

Supplementary Table 2. Data collection and refinement statistics

	T.cruzi PKAR:200-503
**Data collection**	
Space group	P 1 21 1
Cell dimensions	
a, *b*, *c* (Å)	40.85, 89.07, 45.63
a, b, g (°)	90, 98.18,90
Resolution (Å)	40.43 - 1.092 (1.131 - 1.092)*
Completeness (%)	81.8 (41.0) [$]
Multiplicity	3.5 (2.9)
I / s*I* (mean)	11.3 (1.4)
R_{meas}	6.6 (89.5)
CC 1/2	99.9 (60.6)
Wilson B-factor (Å^2)	10.99
**Refinement**	
No. reflections	109259 (5023)
Reflections used for R-free	2000 (92)
R_{work}	15.0 (26.5)
R_{free}	16.0 (30.7)
Average B factor (Å^2)	17.51
R.m.s. deviations	
Bond lengths (Å)	0.010
Bond angles (°)	1.17
Geometry	
Rotamer outliers (%)	0.76
Clashscore	5.54

*Values in parentheses are for highest-resolution shell.
[$] Data are >94% complete for the resolution range of 40-1.46 Å.

Supplementary Methods

Plasmid constructs and genetically engineered trypanosomes

In situ tagging of PKAC1 and PKAC2: Sequences containing the genomic loci of *T. brucei PKAC1* (Tb927.9.11100) and *PKAC2* (Tb927.9.11030) had been isolated from a λ Dash II phage library containing genomic DNA fragments of *T. brucei* AnTat 1.1 prior to the availability of the trypanosome genome sequence[1]. After cloning into pBluescript KS^+, antibiotic resistance genes were inserted to enable selection in trypanosomes. A phleomycin resistance gene with actin UTRs was inserted 172 nucleotides upstream of the *PKAC1* start codon and a neomycin transferase gene with actin UTRs was inserted within the 3'UTR of PKAC2, 1,185 nucleotides downstream of the *PKAC2* stop codon. These plasmids were further modified as needed. To express fusion proteins, a Ty1 or HA epitope tag was included in frame to the N-terminus of the *PKAC1* or *PKAC2* ORF sequence, respectively. For the endogenous expression of a PKAC1 kinase dead mutant, the mutation N153A (AAT:GCT) was introduced by standard cloning methods. For transfection of trypanosomes, the plasmids were digested within flanking regions of the *PKAC* ORFs (*PKAC1*: HindIII/SdaI; *PKAC2*: CalI/BstZ17I) resulting in replacement of the endogenous *PKAC* gene by homologous recombination. Cells were selected in 2 μg ml^{-1} phleomycin or 2 μg ml^{-1} G418, respectively. For overexpression of PKAC3 (Tb927.10.13010), the *PKAC3* ORF fused to an N-terminal Ty1-tag was cloned into pTSARib[HYG][2]. The plasmid was linearized with SphI for transfection and cells were grown in the

presence of 4 μg ml^{-1} hygromycin B. A *T. brucei* cell line inducibly overexpressing PKAR (Tb927.11.4610; AnTat 1.1 GenBank AF182823) was generated by transfection with a plasmid based on plew82[3] containing the *PKAR* ORF fused to a C-terminal Ty1-tag. The plasmid was linearized with NotI for transfection and 2.5 μg ml^{-1} phleomycin was used for selection.

For *in situ* tagging of PKAR with a C-terminal PTP tag (Protein A-TEV-Protein C)[4], the U170k coding sequence of pC-PTP-NEO[4] (kindly provided by A. Bindereif, Giessen) was replaced by the *PKAR* ORF. The plasmid was linearized with XcmI for transfection and cells were selected with 2 μg ml^{-1} G418. For inducible RNAi of *PKAC1/2,* an N-terminal region of the *PKAC1* and *PKAC2* ORFs (nucleotides 30-648; difference between *PKAC1* and *PKAC2* within that region is only three nucleotides with no homology to *PKAC3*) was cloned into the RNAi vector p2t7^{Ti}TAblue[5]. The plasmid was linearized with NotI and transfected into the MiTat 1.2 1313-514 cell line[5], followed by selection with 2 μg ml^{-1} hygromycin B. For inducible RNAi of *PKAC3*, the complete 5'UTR (171 nucleotides) and the N-terminal 295 nucleotides of the *PKAC3* ORF were cloned into p2t7^{Ti}TAblue[5] followed by linearization with NotI, transfection into MiTat 1.2 1313-514[5] and selection with 2 μg ml^{-1} hygromycin B. For RNAi of *PKAR*, the N-terminal fragment of the *PKAR* ORF (nucleotides 1-467) was cloned into the vector p2t7^{Ti}A[6]. The plasmid was linearized with NotI, transfected into the 13-90 cell line[3] and cells were selected in 3 μg ml^{-1} phleomycin. A homozygous *PKAR* deletion mutant was described previously[7]. In order to generate an endogenous single-allele *PKAR* add-back cell line, a pBSK- based plasmid was generated

containing the *PKAR* ORF downstream of a phleomycin resistance gene with aldolase 5'UTR and actin 3'UTR. Flanking *PKAR* 5' and 3'UTRs enabled insertion of the whole cassette into the endogenous *PKAR* locus after digestion with KpnI and XmnI. Cells were selected with 2.5 µg ml^{-1} phleomycin. *T. brucei* cell lines expressing VASP were generated by transgenic expression of the *VASP* ORF amplified from human cDNA (VASP p14/1 in pBSK-[8], Acc. No. Z46389; kindly provided by U. Walter, University of Würzburg Medical Clinic) and cloned into pTSARib[2] after exchange of the original hygromycin resistance cassette against blasticidin or puromycin. These plasmids were linearized with SphI prior to transfection and cells were selected with 4 µg ml^{-1} blasticidin or 0.1 µg ml^{-1} puromycin, respectively. The VASP protein was also expressed with a C-terminal Ty1 tag in the 13-90 cell line[3] using the vector plew82[3] to allow tetracycline inducible expression. Selection was done with 1 µg ml^{-1} phleomycin. This cell line was further transfected with a plasmid allowing hairpin RNAi-mediated repression of the *T. brucei* cAMP-specific phosphodiesterases *PDEB1* and *PDEB2* using the previously published fragment common to both isoforms (nucleotides 1,965-2,201)[9] that was cloned into vector pHD615[PAC] (derivative of pHD615[10] with puromycin resistance gene). The plasmid was linearized with NotI and transfected cells were selected in the presence of 0.1 µg ml^{-1} puromycin. A VASP expressing cell line with homozygous deletion of *pkac3* and RNAi against *PKAC1/2* was generated in *T. brucei* MiTat 1.2 cells expressing a double Tet repressor (pHD1313)[5]. The *pkac3* deletion constructs contained a neomycin or hygromycin resistance gene, respectively, flanked by actin UTRs

and *PKAC3* 5' (794 nucleotides, genomic location: Tb927_10_v5.1: 3,162,016..3,162,810) and 3' stretches (911 nucleotides, genomic location: Tb927_10_v5.1:3,159,790..3,160,700). Linearization for transfection was done using DraIII and PvuI or HindIII, respectively. This cell line was further transfected with a pHD615.PKAC1/2 hairpin RNAi construct (targeting nucleotides 47-501). Selection was performed with 0.2 µg ml^{-1} phleomycin, 2 µg ml^{-1} G418, 2.5 µg ml^{-1} hygromycin B and 0.1 µg ml^{-1} puromycin.

Primer sequences are listed below; cloning strategies are available upon request.

Primers used in this study:

pGEX-4T3.GST-PKAC1(277-334) (expression of GST-PKAC1(277-334) in *E. coli* for antibody production)

C3-AK	cgggatcCCCATTCTTTCGTGGTGC
C3-STOP	ggggtaccgtcgaCTAAAAACCACGGAATG

pGEX-4T3.GST-PKAC3(272-337) (expression of GST-PKAC3(272-337) in *E. coli* for antibody production)

CK16-AK	cgggatcCAAGTTCTACAGTGGGGT
CK16-STOP	ggggtaccgtcgaCTCAGATCCTCGTGTATTCA

pSCTEV3s.PKAC1 / PKAC2 / PKAC3 (expression of PKAC1, C2, C3 in HEK293 cells)

C3-ATG	cgggatccATGACGACAACTCCCAC
C3-STOP	ggggtaccgtcgaCTAAAAACCACGGAATG
34-ATG	cgggatccATGCTGTTGGTGTTACTT
34-STOP	ggggtaccTAAAACCCACGGAACT
CK16-ATG	cgggatccATGAAGTCGGATGGGTG
CK16-STOP	ggggtaccgtcgaCTCAGATCCTCGTGTATTCA

pTy1-PKAC1 (In situ Ty1-tagging of PKAC1)

Ty1_up	CGTGAGGTCCATACTAACCAGGACCCACTTGAC
Ty1_rev	CACGGTCAAGTGGGTCCTGGTTAGTATGGACCT

pHA-PKAC2.NEO (In situ HA-tagging of PKAC2)

HA-SalI-TbPKAC2 upp	AGTTCGAGTCGACGTACCCATACGACGTCCCAG ACTACGCTGAACCGCAAACGT
TbPKAC2-CTerm.lower	CTTTACGAGATCCCGAGC

p∆PKAC1.BSD (deletion of single PKAC1 allele)

A/B 3´ flank forward	GCTCTAGAGCGTTGGGCACGCTGA
A 3´flank rev	GGAATTCCGATGTAACGATGGGAA

pTy1-PKAC1dead (expression of PKAC1 N153A (dead mutant))

PKAC1seq.u.19/7/01	CTAATGGAGTTGTCACACCCC
PKA_N153->A.l.Eco31	ACTGAGGTCTCTGAGCCTCAGGTTTCAAGTCAC
PKA-N153->A.u.Eco31	ACTGAGGTCTCGGCTCTGCTACTTGATGGGAAG
PKAC1.seq.l.19/7/01	GCAGTGAAAACCAAGAAAGGG

pTSARibTy1-PKAC3 (ectopic expression of Ty1-PKAC3)

PKAC3-HindIIIty1ATG.u.	CTGGAAGCTTATGACCGGTGAGGTCCATACTAAC CAGGACCCACTTGACAAGTCGGATGGGTGCTTG
PKAC3-BamHI.l.	CTGGGGATCCTCAGATCCTCGTGTATTC

plew82.PKAR-TY (Inducible, ectopic expression of PKAR-Ty1)

PKAR XhoI fw	TGTGGATGAGCTCGAGTTCCTTAACAATCA
PKAR TyBglII 2	CGCAGATCTCTAGTCAAGTGGATCCTGGGTTAGT ATGGACCTCCTTCCTCCCCTCTGCCCTTA

pC-Neo.PKAR-PTP (In situ PTP-tagging of PKAR)

PKAR-upper-ApaI	CTAATGGGCCCATGTCTGAAAAGGGAACATCG
PKAR-lower-NotI	ATAATGCGGCCGCGTCTTCCTCCCCTCTGCCCT

p2T7TAblue PKAC1/2 RNAi

PKAC1/2 RNAi forward	CCGCTCGAGTCTAGAATTTGT
PKAC1/2 RNAi reverse	CCCAAGCTTGAATTCATACAG

p2T7TAblue PKAC3 RNAi

PKAg 5' BamHI	gaggatccAGAAACGGTAACAGGACAGCGTG
PKAg 3' XhoI lo	tactcgagGAGGAGGTACAATCGGTCA

p2T7 PKAR RNAi

PKAR 5' end	TCCATGTCTGAAAAGGGAACATCGTTAAACC
PKAR 3' BamHI	AAAGGGATCCAATGTATATTTTACGAGCCACC

pBSK.PKAR (PKAR in situ rescue)

PKAR-3'UTR-upper-Bam	ACTACGGATCCGGGCAGAGGGGAGGAAGTAG
PKAR-3'UTR-lower-Xba	GTTAAGTCTAGAAAACAGAACCTCCCGAGCAC
5'RUTR-KpnI.U1	atattggtaccTTTACGGCAAGGAATCGTTC
5'RUTR-EcoRV.L1	attaagatatcAACGATGTTCCCTTTTCAGAC

pTSARib.HYG.VASP

VASP-HindIII.u	ACGTAAGCTTATGAGCGAGACGGTCAT
VASP-BamHI.l	ACGTGGATCCTCAGGGAGAACCCCGCTT

pTSARib.PAC.VASP (exchange of resistance cassette from pTSARib.HYG to PAC)

Puro upper 1	TAGTGCTAGCGGGCACAGCAAGGTCT
Puro lower 1	ACTATTTCAATCATGTCGA

plew82.VASP

pTSARib.VASP_up1	ATCGGTGGCGGCCGGATGAGCGAGACGGTCATCTGTT

pTSARib.VASP_lp1	AGCAGGATCCTCAGTCAAGTGGGTCCTGGTTAG TATGGACCTCGGGAGAACCCCGCTTCCTC

pHD615.PDEB1/2 RNAi

PDEB1/2 RNAi 5S	TGTTAAGCTTCTTCTCTATGTTTGCTGGC
PDEB1/2 RNAi 3S	CAATCTCGAGGCCACACGGAAAACGAATC
PDEB1/2 RNAi 3AS	TGTTGGATCCCTTCTCTATGTTTGCTGGC
PDEB1/2 RNAi 5AS	ACGCCTCGAGTGGTACGCGTCCTGAATAT

pHD615.PKAC1/2 RNAi

PKAC1/2 3'S	TCCCTctcgagCAAGTAGCAGATTCTCAGG
PKAC1/2 5'AS	CTGGTggatccCAACTGTTTACCAAGCCTG
PKAC1/2 3'AS	GGTACctcgagTCACCTTCTTAGCAAAACC
PKAC1/2 5'S 2	CAAAAaagcttAGCAGGGGGGAGTATTATG

pET-DUET TbPKAR(199-499) (expression of TbPKAR(199-499) in *E. coli*)

TbPKAR199-499_FW	gccaGGATCCGgaaaacctgtattttcagGGATCTGGCCG GAAGCGACG
TbPKAR199-499_RV	cattatgcggccGCttaCTTCCTCCCCTCTGCCCT

pET-DUET TcPKAR(200-503) (expression of TcPKAR(200-503) in *E. coli)*

TcPKAR200-503_FW	agccaGGATCCGgaaaacctgtattttcagGGATCTGGCAG AAACCGCCGCC
TcPKAR200-503_RV	cattatgcggccGCTTATACATCATCCACGTACGATG

Co-expression of PKAR-10xHis and Strep-PKAC1 in Lexsy (vectors pLEXSY_I-ble3, pLEXSY_I-neo3)

5' PKAR SalI	ATGCATCTCGAGATGTCTGAAAAGGGAACATCG
3' PKAR NotI	TATATAATAGTTTAGCGGCCGCTCAGTGGTGGTGATGGTGGTGGT GGTGGTGATGGCTACCGCCCTTCCTCCCCTCTGCCCTTAA
5' Strep BamHI	CGCGGATCCATGGCTTCGGCTTGGAGCCAC
PKAC1 Rev NotI	ACTATGCAATAGTTTAGCGGCCGCCTAAAAACCAC GGAATGCAAC
5' Ble BamHI	GCCGGATCCACCATGGCCAAGTTGACCAGT
3' Ble SpeI	CTAGAACTAGTTCAGTCCTGCTCCTCGGCCA

Co-expression of human PKARIalpha and mouse PKACalpha (in pETDuet-1)

5' strep (NdeI) Mm Ca	ttGGAATTCCATATGGCTTCGGCTtggagccacccgcagttc gaaaaaGCTGGCAACGCCGCCGCCGCCAAGAAGGGC
3' (AvrII) MmCα	ttcTGCCTAGGCTAAAACTCAGTAAACTCCTTGCC
6H.tev/HsPKARIaf	GCCAGGATCCGGAAAACCTGTATTTTCAGGGATCTAT GGAGTCTGGCAGTA
6H.tev/HsPKARIar	attatgcggccGCTCAGACAGACAGTGA

Generation of polyclonal antibodies

Rabbits were immunized by Eurogentec with 500 µg truncated GST-PKAC fusion protein (PKAC1(277-334), PKAC3(272-337)) purified from *E. coli* BL21DE3, followed by further boosts with 500 µg antigen. Full-length His_6-fusion proteins expressed in *E. coli* M15 (Qiagen) and purified using Ni-NTA columns (Qiagen) were used for affinity purification of the antibodies according to the method of Olmsted [11]. Isoform specificity of affinity-purified sera was established by Western blot analysis upon expression of full-length *T. brucei* PKAC1, PKAC2 or PKAC3 in human 293 cells (obtained from P. Matthias, Basel) (Supplementary Fig. 1a). No cross-reactivity with human catalytic subunits was observed. The antibody raised against PKAC1 could not discriminate between the highly similar isoforms PKAC1 and PKAC2.

Protein expression in HEK293 cells

The *PKAC1*, *PKAC2* and *PKAC3* ORFs were cloned into pSCTEV3s[12] for expression in HEK293 cells as GST-fusion proteins. Cultivation and transfection was performed as described in Vassella et al.[13]. For antibiotic selection, 300 µg ml^{-1} hygromycin B and 250 µg ml^{-1} G418 were added to the culture medium.

Southern blot

Total genomic DNA was isolated by phenol/chloroform extraction. Restriction enzyme digests of genomic DNA were analysed by Southern blot with digoxigenine labelled probes (DIG DNA Labeling and Detection Kit, Roche).

Western blot

For Western blot analysis[14], the primary antibodies anti-PKAR[7] (1:500), anti-PKAC1/2 (1:500-1:1,000), anti-PKAC3 (1:500), anti-metacaspase 4 (MCA4[15], 1:800, kindly provided by J. Mottram, University of York), anti-Ty1[16] (1:300-1:1,000), anti-HA (clone 12CA5; undiluted hybridoma), anti-HSP60[17] (1:10,000), anti-PFR-A/C[18] (1:2,000), anti-VASP (1:5,000; ImmunoGlobe, catalogue number IG-731), and anti-Phospho-(Ser/Thr) PKA substrate antibody (1:1,000; Cell Signalling Technologies, catalogue number 9621) were used. Labelled secondary antibodies IRDye® 800CW goat anti-mouse (LICOR, catalogue number 925-32210, 1:5000) and IRDye® 680LT goat anti-rabbit (LICOR, catalogue number 925-69021, 1:5000) or Alexa Fluor® 680 goat anti-rabbit (ThermoFisher, catalogue number A27042, 1:5000) were used for detection using the Odyssey™ IR fluorescence scanning system. Signals of *T. brucei* PKA subunit proteins, metacaspase 4 or phospho-RXXS/T-containing proteins were normalized to the PFR-A/C or Hsp60 loading control after automatic subtraction of the background values (Median Left/Right method) using the Odyssey software (LI-COR). For the in vivo PKA reporter assay, the signals of the upper band (phosphorylated) and the lower band (non-phosphorylated) of VASP were quantified, and the % phosphorylated calculated as (phosphorylated VASP / (phosphorylated + non-phosphorylated VASP)) × 100.

Microscopic analysis

For microscopic analysis, 5×10^5 trypanosome cells were spread on glass slides and fixed overnight in methanol at -20°C. Cellular DNA was visualized with 4′,6-diamidino-2-phenylindole (DAPI; 1 µg ml^{-1}). Image acquisition was performed with a motorized Zeiss Axiophot2 wide-field microscope equipped with a Zeiss 63×/1.4 NA Oil DIC objective, a 1.0×-2.5× optovar, and a Princeton Instruments Micromax cooled (-15°C) slow scan CCD camera (Kodak KAF-1400 CCD chip).

Cyclic AMP measurement

Prior to lysis, all steps were performed identical to the in vivo PKA reporter assay. 2×10^7 cells were then pipetted into 5 ml of boiling water and incubated for 5 minutes, followed by lyophilisation. cAMP concentrations were determined using the Cyclic AMP XP® Assay Kit (NEB). Three independent experimental series were analysed in duplicates.

Cell viability assay

The viability of *T. brucei* BSF cells upon treatment with various compounds was assessed using the Alamar blue assay as published[19] with the following modifications: initial seeding density was reduced from 1×10^5 to 5×10^3 trypanosomes ml^{-1}, the growth period was extended from 48 h to 72 h, and the subsequent incubation with 0.5 mM resazurin sodium salt was reduced from 24 h to 4 h. The cytotoxicity of compounds used in this study was determined by plotting the percentage of survival against the compound concentration followed

by calculation of the EC_{50} by non-linear regression analysis using an equation for a sigmoidal dose-response curve with variable slope (GraphPad Prism 7.0).

Recombinant expression of human PKARIα-PKACα holoenzyme

Human *PKARIα* (with an N-terminal 6×His-tag followed by a TEV cleavage site) was amplified by PCR from the plasmid pRSETb-hRIα kindly provided by F. Herberg, University of Kassel. Mouse *PKACα* (with an N-terminal Strep-tag) was amplified by PCR from mouse c2c12 cDNA kindly provided by H. Leonhard, LMU Munich. The full length ORFs (amplified with primers introducing the respective epitope tag) were cloned into pETDuet-1 (Novagene) and co-expressed in *E. coli* strain APE304[20] (kindly provided by C. Dehio, University of Basel). This strain is devoid of adenylate cyclases and thereby does not produce cAMP; this facilitated purification of an intact holoenzyme complex. Details on primer sequences and cloning strategies are available upon request. Purification of the holoenzyme complex was performed according to the protocol given for the *T. brucei* PKA holoenzyme.

Protein crystallization

N-terminally truncated *Trypanosoma cruzi* PKAR(200-503) (TritrypDB Tc00.1047053506227.150) was cloned into pETDuet-1 (Novagene) with an N-terminal 6×His-tag in tandem with a TEV protease recognition site and transformed into *E. coli* Rosetta (DE3). Bacteria were grown at 37°C until reaching an OD_{600} of 0.8, followed by overnight induction with 0.1 mM IPTG at

16°C. The protein was purified via affinity chromatography on a Ni-NTA column, followed by TEV protease cleavage of the 6×His-tag and dialysis against 50 mM HEPES pH 7.5, 50 mM NaCl and concentration by ultrafiltration. The purified protein (≈10 mg ml^{-1}) was denatured by addition of solid urea (final concentration = 8M) and subjected to a small-scale gel filtration using a PD10 column to remove any bound metabolites derived from *E. coli*. The flow through containing the denatured, ligand-free protein was diluted to 1 mg ml^{-1} in 8 M urea, 50 mM HEPES pH 7.5 buffer (final volume = 10 ml) and dialyzed overnight at 4°C against 1 l of refolding buffer (50 mM Tris pH 8.5, 240 mM NaCl, 10 mM KCl, 2 mM $MgCl_2$, 2 mM $CaCl_2$, 0.4 M sucrose, 1 mM DTT, 1% DMSO). 1 mM of 7-CN-7-C-Ino was added to the denatured protein prior to dialysis. The refolded protein was concentrated to 1 ml and subjected to size exclusion chromatography on a Superdex 200 Increase 10/300 GL column in elution buffer (50 mM HEPES pH 7.5, 50 mM NaCl, 1% DMSO). The eluted protein fraction was concentrated to 10 mg ml^{-1} and stored at 4°C with a 10-fold molar excess of 7-CN-7-C-Ino. Crystals grew after one week at 4°C in a mixture of 100 nl protein and 100 nl crystallization solution (17% PEG 6,000 and 0.2 M calcium acetate) using the sitting drop vapour diffusion method. Prior to flash cooling in liquid nitrogen, the crystals were briefly soaked in mother liquor containing 30% (v/v) ethylene glycol. The X-ray diffraction data were collected at the Swiss Light Source beam line PXIII with a wavelength of 0.9794 Å at a temperature of 100 K and processed using the program XDS[21]. The structure of TcPKAR was determined by molecular replacement using the *T. brucei* PKAR structure (unpublished) as a

search model in Phaser[22] as implemented in the program PHENIX[23]. The model was completed by iterative cycles of manual building in Coot[24] and refinement in PHENIX. The final model includes residues 211–501 for which a continuous electron density was observed. The Ramachandran plot predicts 98.7% of the angles to be in a favoured geometry and 1.3% to be found in an allowed geometry. No outliers were detected. For refinement statistics see S2 Table. The data are >98% complete in the range of 40-2.3 Å and 94.1% complete in the 1.64-1.46 Å resolution shell. By inclusion of higher resolution shells with lower completeness, we achieve an effective resolution of 1.15-1.2 Å. The coordinates were deposited in the Protein Data Bank under the code PDB 6FTF [http://dx.doi.org/10.2210/pdb6FTF/pdb].

Molecular docking

For the docking experiment, the software used was Glide[25] as implemented in Maestro (Schrödinger). The ligands were built manually and prepared using LigPrep (Schrödinger). The docking strategy used was core docking using the cognate ligands as a reference while the docking mode chosen was SP (Standard Precision). In the CNB-A site, the grid was built using glutamates E310 and E312 and the water molecules w75 and w442, which were the ones in closest proximity with the ligand in the crystal structure. In the CNB-B site, the docking grid is composed by glutamate E436 and the structural water w07. Poses were analysed by visual inspection using Pose Viewer (Schrödinger) and ranked according to their Glide E-model docking score.

Quantitative proteomics

Trypanosomes treated or not with 2 µM 7-CN-7-C-Ino (time course: 0 h, 6 h, 12 h) were washed twice in phosphate buffered saline (PBS) and lysed in Laemmli sample buffer to a concentration of 2.5×10^5 cells μl^{-1}. Protein concentration was determined by Colloidal Coomassie Blue staining and densitometry. 20 µg of protein were loaded on a 10% acrylamide SDS-PAGE gel. Migration was stopped when samples had entered the resolving gel and proteins were visualized by Colloidal Coomassie Blue staining. Each SDS-PAGE band was cut into 1 mm × 1 mm gel pieces and further processed as described previously[26]. Online nanoLC-MS/MS analyses were performed using an Ultimate 3000 RSLC Nano-UPHLC system (Thermo Scientific, USA) coupled to a nanospray Q-Exactive hybrid quadrupole-Orbitrap mass spectrometer (Thermo Scientific, USA). 1 µg of each peptide extract was loaded on a 300 µm ID x 5 mm PepMap C_{18} precolumn (Thermo Scientific, USA) at a flow rate of 20 $\mu l\ min^{-1}$. After a 3 min desalting step, peptides were separated on a 75 µm ID x 25 cm C_{18} Acclaim PepMap® RSLC column (Thermo Scientific, USA) with a 4-40% linear gradient of solvent B (0.1% formic acid in 80% ACN) in 108 min. The separation flow rate was set at 300 $nl\ min^{-1}$. The mass spectrometer operated in positive ion mode at a 1.8 kV needle voltage. Data was acquired using Xcalibur 3.1 software in a data-dependent mode. MS scans (m/z 300-1,600) were recorded at a resolution of R = 70,000 (@ m/z 200) and an AGC target of 3×10^6 ions collected within 100 ms. Dynamic exclusion was set to 30 s and top 12 ions were selected from fragmentation in HCD mode. MS/MS scans with a target value of 1×10^5 ions

were collected with a maximum fill time of 100 ms and a resolution of R = 17,500. Additionally, only +2 and +3 charged ions were selected for fragmentation. Other settings were as follows: no sheath and no auxiliary gas flow, heated capillary temperature (200°C), normalized HCD collision energy of 27 eV and an isolation width of 2 m/z.

Raw spectra were analysed using MaxQuant version 1.6.1.0[27], which incorporates the Andromeda search engine, using default settings and the *Trypanosoma brucei* TriTrypDB-36_TbruceiTREU927 protein database. Carbamidomethyl-cystein was set as fixed modification and oxidation (M), acetylation (protein N-terminal, K) and deamidation (N, Q) as dynamic modifications. The MaxQuant output was loaded into Perseus version 1.6.0.7[28] and filtered to exclude proteins 'only identified by site', reverse hits and potential contaminants. Stringent selection criteria were applied in order to exclude potential outliers: only proteins that were identified with LFQ values greater than zero in all wild type samples at time points 0 and 6 hours or 6 and 12 hours were included in the following analysis. Moreover, the LFQ values of these proteins in the *pkar* knock out samples had to be either all equal to zero or all unequal zero. The LFQ values of the remaining proteins were $\log_2$ transformed and missing values were imputed from normal distributions. The statistical significance of changes in protein abundance was analysed by a two-sided Welch t-test for LFQ values +/- compound treatment (6 h, 12 h) in wild type trypanosomes as well as in the *PKAR* deletion mutant followed by visualization of all comparisons by volcano plots. The raw and processed mass spectrometry proteomics data have

been deposited to the ProteomeXchange Consortium (http://proteomecentral.proteomexchange.org) via the PRIDE partner repository[29] with the dataset identifier PXD009073 [http://www.ebi.ac.uk/pride/archive/projects/PXD009073].

Supplementary References

1 Kramer, S., Klockner, T., Selmayr, M. & Boshart, M. Interstrain sequence comparison, transcript map and clonal genomic rearrangement of a 28 kb locus on chromosome 9 of *Trypanosoma brucei*. *Molecular and biochemical parasitology* **151**, 129-132, doi:10.1016/j.molbiopara.2006.10.004 (2007).

2 Xong, H. V. *et al.* A VSG expression site-associated gene confers resistance to human serum in *Trypanosoma rhodesiense*. *Cell* **95**, 839-846 (1998).

3 Wirtz, E., Leal, S., Ochatt, C. & Cross, G. A. A tightly regulated inducible expression system for conditional gene knock-outs and dominant-negative genetics in *Trypanosoma brucei*. *Molecular and biochemical parasitology* **99**, 89-101 (1999).

4 Schimanski, B., Nguyen, T. N. & Gunzl, A. Highly efficient tandem affinity purification of trypanosome protein complexes based on a novel epitope combination. *Eukaryotic cell* **4**, 1942-1950, doi:10.1128/EC.4.11.1942-1950.2005 (2005).

5 Alibu, V. P., Storm, L., Haile, S., Clayton, C. & Horn, D. A doubly inducible system for RNA interference and rapid RNAi plasmid construction in *Trypanosoma brucei*. *Molecular and biochemical parasitology* **139**, 75-82, doi:10.1016/j.molbiopara.2004.10.002 (2005).

6 LaCount, D. J., Barrett, B. & Donelson, J. E. *Trypanosoma brucei* FLA1 is required for flagellum attachment and cytokinesis. *J Biol Chem* **277**, 17580-17588, doi:10.1074/jbc.M200873200 (2002).

7 Bachmaier, S. *et al.* Protein kinase A signaling during bidirectional axenic differentiation in *Leishmania*. *International journal for parasitology* **46**, 75-82, doi:10.1016/j.ijpara.2015.09.003 (2016).

8 Haffner, C. *et al.* Molecular cloning, structural analysis and functional expression of the proline-rich focal adhesion and microfilament-associated protein VASP. *The EMBO journal* **14**, 19-27 (1995).

9 Oberholzer, M. *et al.* The *Trypanosoma brucei* cAMP phosphodiesterases TbrPDEB1 and TbrPDEB2: flagellar enzymes that are essential for parasite virulence. *FASEB* **21**, 720-731 (2007).

10 Biebinger, S., Wirtz, L. E., Lorenz, P. & Clayton, C. Vectors for inducible expression of toxic gene products in bloodstream and procyclic *Trypanosoma brucei*. *Molecular and biochemical parasitology* **85**, 99-112 (1997).

11 Olmsted, J. B. Affinity purification of antibodies from diazotized paper blots of heterogeneous protein samples. *J Biol Chem* **256**, 11955-11957 (1981).

12 Matthias, P., Muller, M. M., Schreiber, E., Rusconi, S. & Schaffner, W. Eukaryotic expression vectors for the analysis of mutant proteins. *Nucleic acids research* **17**, 6418 (1989).

13 Vassella, E. *et al.* Deletion of a novel protein kinase with PX and FYVE-related domains increases the rate of differentiation of *Trypanosoma brucei*. *Molecular microbiology* **41**, 33-46 (2001).

14 Salmon, D. *et al.* Cytokinesis of *Trypanosoma brucei* bloodstream forms depends on expression of adenylyl cyclases of the ESAG4 or ESAG4-like subfamily. *Molecular microbiology* **84**, 225-242, doi:10.1111/j.1365-2958.2012.08013.x (2012).

15 Proto, W. R. *et al. Trypanosoma brucei* metacaspase 4 is a pseudopeptidase and a virulence factor. *J Biol Chem* **286**, 39914-39925, doi:10.1074/jbc.M111.292334 (2011).

16 Bastin, P., Bagherzadeh, Z., Matthews, K. R. & Gull, K. A novel epitope tag system to study protein targeting and organelle biogenesis in *Trypanosoma brucei*. *Molecular and biochemical parasitology* **77**, 235-239 (1996).

17 Bringaud, F. *et al.* Molecular characterization of the mitochondrial heat shock protein 60 gene from Trypanosoma brucei. *Molecular and biochemical parasitology* **74**, 119-123 (1995).

18 Kohl, L., Sherwin, T. & Gull, K. Assembly of the paraflagellar rod and the flagellum attachment zone complex during the *Trypanosoma brucei* cell cycle. *The Journal of eukaryotic microbiology* **46**, 105-109 (1999).

19 Gould, M. K. *et al.* Cyclic AMP effectors in African trypanosomes revealed by genome-scale RNA interference library screening for resistance to the phosphodiesterase inhibitor CpdA. *Antimicrobial agents and chemotherapy* **57**, 4882-4893, doi:10.1128/AAC.00508-13 (2013).

20 Pulliainen, A. T. *et al.* Bacterial effector binds host cell adenylyl cyclase to potentiate Galphas-dependent cAMP production. *Proceedings of the National*

Academy of Sciences of the United States of America **109**, 9581-9586, doi:10.1073/pnas.1117651109 (2012).

21 Kabsch, W. XDS. *Acta crystallographica. Section D, Biological crystallography* **66**, 125-132, doi:10.1107/s0907444909047337 (2010).

22 McCoy, A. J. *et al.* Phaser crystallographic software. *Journal of applied crystallography* **40**, 658-674, doi:10.1107/s0021889807021206 (2007).

23 Adams, P. D. *et al.* PHENIX: a comprehensive Python-based system for macromolecular structure solution. *Acta Crystallographica Section D: Biological Crystallography* **66**, 213-221, doi:10.1107/S0907444909052925 (2010).

24 Emsley, P. & Cowtan, K. Coot: model-building tools for molecular graphics. *Acta crystallographica. Section D, Biological crystallography* **60**, 2126-2132, doi:10.1107/s0907444904019158 (2004).

25 Friesner, R. A. *et al.* Glide: a new approach for rapid, accurate docking and scoring. 1. Method and assessment of docking accuracy. *Journal of medicinal chemistry* **47**, 1739-1749, doi:10.1021/jm0306430 (2004).

26 Allmann, S. *et al.* Triacylglycerol Storage in Lipid Droplets in Procyclic *Trypanosoma brucei*. *PLOS ONE* **9**, e114628, doi:10.1371/journal.pone.0114628 (2014).

27 Cox, J. & Mann, M. MaxQuant enables high peptide identification rates, individualized p.p.b.-range mass accuracies and proteome-wide protein quantification. *Nature biotechnology* **26**, 1367-1372, doi:10.1038/nbt.1511 (2008).

28 Tyanova, S., Temu, T. & Sinitcyn, P. The Perseus computational platform for comprehensive analysis of (prote)omics data. **13**, 731-740, doi:10.1038/nmeth.3901 (2016).

29 Vizcaino, J. A. *et al.* 2016 update of the PRIDE database and its related tools. *Nucleic acids research* **44**, D447-456, doi:10.1093/nar/gkv1145 (2016).
