# Supplementary material for: Nucleoside analogue activators of cyclic AMP-independent protein kinase A of Trypanosoma
Source: Nat Commun. 2019 Mar 29;10:1421. doi: 10.1038/s41467-019-09338-z (PMC6440977; doi:10.1038/s41467-019-09338-z)
Supplement: Supplementary file 3 — Reporting Summary [file 41467_2019_9338_MOESM3_ESM.pdf]

nature research

Corresponding author(s): Michael Boshart

Reporting Summary

Nature Research wishes to improve the reproducibility of the work that we publish. This form provides structure for consistency and transparency in reporting. For further information on Nature Research policies, see Authors & Referees and the Editorial Policy Checklist.

Statistical parameters

When statistical analyses are reported, confirm that the following items are present in the relevant location (e.g. figure legend, table legend, main text, or Methods section).

n/a	Confirmed	
☐	☒	The exact sample size (*n*) for each experimental group/condition, given as a discrete number and unit of measurement
☐	☒	An indication of whether measurements were taken from distinct samples or whether the same sample was measured repeatedly
☐	☒	The statistical test(s) used AND whether they are one- or two-sided<br>*Only common tests should be described solely by name; describe more complex techniques in the Methods section.*
☒	☐	A description of all covariates tested
☐	☒	A description of any assumptions or corrections, such as tests of normality and adjustment for multiple comparisons
☐	☒	A full description of the statistics including central tendency (e.g. means) or other basic estimates (e.g. regression coefficient) AND variation (e.g. standard deviation) or associated estimates of uncertainty (e.g. confidence intervals)
☒	☐	For null hypothesis testing, the test statistic (e.g. *F*, *t*, *r*) with confidence intervals, effect sizes, degrees of freedom and *P* value noted<br>*Give P values as exact values whenever suitable.*
☒	☐	For Bayesian analysis, information on the choice of priors and Markov chain Monte Carlo settings
☒	☐	For hierarchical and complex designs, identification of the appropriate level for tests and full reporting of outcomes
☒	☐	Estimates of effect sizes (e.g. Cohen's *d*, Pearson's *r*), indicating how they were calculated
☐	☒	Clearly defined error bars<br>*State explicitly what error bars represent (e.g. SD, SE, CI)*

Our web collection on statistics for biologists may be useful.

Software and code

Policy information about availability of computer code

Data collection	All software used in this study for data collection are mentioned in the manuscript and are either commercially available or open source.
Data analysis	All software used in this study for data analysis are mentioned in the manuscript and are either commercially available or open source.

For manuscripts utilizing custom algorithms or software that are central to the research but not yet described in published literature, software must be made available to editors/reviewers upon request. We strongly encourage code deposition in a community repository (e.g. GitHub). See the Nature Research guidelines for submitting code & software for further information.

Data

Policy information about availability of data

All manuscripts must include a data availability statement. This statement should provide the following information, where applicable:

- Accession codes, unique identifiers, or web links for publicly available datasets
- A list of figures that have associated raw data
- A description of any restrictions on data availability

The coordinates of the T. cruzi PKAR crystal structure bound to 7-CN-7-C-Ino have been deposited in the Protein Data Bank under the code PDB 6FTF [http://dx.doi.org/10.2210/pdb6FTF/pdb]. The phosphoproteome and proteome datasets are available in the PRIDE partner repository with the dataset identifiers PXD012245 [http://www.ebi.ac.uk/pride/archive/projects/PXD012245] and PXD009073 [http://www.ebi.ac.uk/pride/archive/projects/PXD009073], respectively.

April 2018

Genome sequence and annotation information was obtained from TritrypDB (http://www.tritrypDB.org). Human PKA substrates and phosphorylation motifs were retrieved from the PhosphoSitePlus database (https://www.phosphosite.org). Gene ontology (GO) enrichment analysis was visualized using Revigo (http://revigo.irb.hr). The motif discovery tool MoMo implemented in the MEME suite (http://meme-suite.org/) was used for unbiased motif discovery in the phosphoproteome dataset. The source data underlying Fig. 1c, d, Fig. 2a-h, Fig. 3a-c, Fig. 5a, c-e, Table 1 and Suppl. Fig. 1b, Suppl. Fig. 2, Suppl. Fig. 3a-d, f-i, Suppl. Fig. 4a-h, Suppl. Fig. 8a-c, Suppl. Fig. 9a-c are provided as Source Data file.

Field-specific reporting

Please select the best fit for your research. If you are not sure, read the appropriate sections before making your selection.

☒ Life sciences ☐ Behavioural & social sciences ☐ Ecological, evolutionary & environmental sciences

For a reference copy of the document with all sections, see nature.com/authors/policies/ReportingSummary-flat.pdf

Life sciences study design

All studies must disclose on these points even when the disclosure is negative.

Sample size	No statistical methods were used to determine sample sizes.
Data exclusions	No data were excluded.
Replication	All replicates are reported in the manuscript.
Randomization	n/a
Blinding	Blinding was not implemented in this study.

Reporting for specific materials, systems and methods

Materials & experimental systems

n/a	Involved in the study	
☒	☐	Unique biological materials
☐	☒	Antibodies
☐	☒	Eukaryotic cell lines
☒	☐	Palaeontology
☒	☐	Animals and other organisms
☒	☐	Human research participants

Methods

n/a	Involved in the study	
☒	☐	ChIP-seq
☒	☐	Flow cytometry
☒	☐	MRI-based neuroimaging

Antibodies

Antibodies used

anti-TbPKAR: polyclonal rabbit antibody detecting T. brucei PKAR, described by Bachmaier et al. 2016, Int. J. Parasitology 46, 75ff
anti-TbPKAC1/2 and anti-TbPKAC3: polyclonal rabbit antibodies detecting T. brucei PKAC1/2 or PKAC3, respectively
anti-MCA4: polyclonal chicken antibody detecting T. brucei MCA4, described by Proto et al. 2011 JBC 286, 39914ff; provided by J. Mottram
anti-Ty1: monoclonal mouse antibody detecting the 10-amino acid Ty1 epitope tag; described by Bastin et al. 1996, Mol Biochem Parasitol. 77, 235ff; provided by K. Gull
anti-HA: monoclonal mouse antibody detecting the HA epitope tag; commercial clone 12CA5, Roche
anti-HSP60: monoclonal mouse antibody detecting T. brucei Hsp60; described by Bringaud et. al 1995, Mol Biochem Parasitol 71, 65ff and Bringaud et al. 1995, Mol Biochem Parasitol 74(1):119ff; provided by F. Bringaud
anti-PFRA/C: monoclonal mouse antibody detecting T. brucei PFR-A/C; described by Kohl et al. 1999, J. Euk Microbiol 46, 105ff; provided by L. Kohl and P. Bastin
anti-VASP: commercial, ImmunoGlobe, Cat. No. IG-731, lot 1703
anti-Phosho-(Ser/Thr) PKA substrate antibody: commercial, Cell Signaling Technologies, Cat. No. 9621, lot 13

Validation

anti-TbPKAR: validated for specific detection of T. brucei PKAR by Western blot analysis of PKAR knock out cells by Bachmaier et al. 2016, Int. J. Parasitology 46, 75ff
anti-TbPKAC1/2 and anti-TbPKAC3: specificity for T. brucei PKAC1/2 or PKAC3, respectively, was proven by Western blot using recombinantly expressed PKAC1, C2 or C3 (see Supplementary Fig. 1a). T. brucei cell lines with knock down of PKAC1/2 or PKAC3, respectively, provide additional validation (Supplementary Fig. 2).
anti-MCA4: validated for specific detection of T. brucei MCA4 by Western blot analysis of MCA4 knock out and knock down cells

by Proto et al. 2011 JBC 286, 39914ff
anti-Ty1: validated for specific detection of Ty1-tagged proteins in T. brucei by Bastin et al. 1996, Mol Biochem Parasitol. 77, 235ff
anti-HA: Western blot detection of a recombinant protein containing the HA epitope (validation done by Roche); used for detection of HA-tagged proteins in T. brucei by many reports, e.g. Kelly et al. 2007, Mol Biochem Parasitol. 154, 103ff; Saada et al. 2014, Eukaryot. Cell, 13(8), 1064ff
anti-HSP60: validated for specific detection of T. brucei (and L. tarentolae) Hsp60 by Western blot analysis of recombinantly expressed Hsp60 and on trypanosome lysates by Bringaud et. al 1995, Mol Biochem Parasitol 74(1), 119ff
anti-PFRA/C: validated for specific detection of T. brucei PFR-A/C by Western blot and immunofluorescence by Kohl et al. 1999, J. Euk Microbiol 46, 105ff; provided by L. Kohl and P. Bastin
anti-VASP: validated for detection of recombinantly expressed VASP in T. brucei in this study, see Supplementary Fig. 3e
anti-Phosho-(Ser/Thr) PKA substrate antibody: validated for detection of phospho-RXXS/T sites in T. brucei by Western blot and mass spectrometry in this study, see Fig. 5

Eukaryotic cell lines

Policy information about cell lines

Cell line source(s)	All T. brucei brucei cell lines are derived in the laboratory from T. brucei brucei stock Lister 427 clone MiTat 1.2, originally obtained from G. Cross, NY (cited in Methods).<br>HEK293 cells were obtained from P. Matthias, Basel.
Authentication	T. brucei brucei stock Lister 427 clone MiTat 1.2: staining with VSG 221 antibody, growth properties
Mycoplasma contamination	n/a
Commonly misidentified lines<br>(See ICLAC register)	n/a
