# Supplementary material for: Nucleoside analogue activators of cyclic AMP-independent protein kinase A of Trypanosoma
Source: Nat Commun. 2019 Mar 29;10:1421. doi: 10.1038/s41467-019-09338-z (PMC6440977; doi:10.1038/s41467-019-09338-z)
Supplement: Supplementary file 4 — Description of Additional Supplementary Files [file 41467_2019_9338_MOESM4_ESM.pdf]

Description of Additional Supplementary Files

File Name: Supplementary Data 1
Description: Phosphopeptides significantly regulated upon 7-CN-7-C-Ino treatment (FDR ≤ 0.05, s_0 = 2).

File Name: Supplementary Movie 1
Description: Structural Alignment *T. cruzi* PKAR(200-503) and bovine PKARIα(92-308)

File Name: Supplementary Movie 2
Description: Comparison of linking helical element in *T. cruzi* PKAR(200-503) and bovine PKARIα(92-308)

File Name: Supplementary Movie 3
Description: *T. cruzi* PKAR CNB-A with bound 7-CN-7-C-Ino

File Name: Supplementary Movie 4
Description: *T. cruzi* PKAR CNB-B with bound 7-CN-7-C-Ino

File Name: Supplementary Movie 5
Description: Steric clash between cAMP and kinetoplastid-specific glutamates in the binding pockets of *T. cruzi* PKAR
